# Generation and Characterization of a SARS-CoV-2-Susceptible Mouse Model Using Adeno-Associated Virus (AAV6.2FF)-Mediated Respiratory Delivery of the Human ACE2 Gene

**DOI:** 10.3390/v15010085

**Published:** 2022-12-28

**Authors:** Nikesh Tailor, Bryce M. Warner, Bryan D. Griffin, Kevin Tierney, Estella Moffat, Kathy Frost, Robert Vendramelli, Anders Leung, Marnie Willman, Sylvia P. Thomas, Yanlong Pei, Stephanie A. Booth, Carissa Embury-Hyatt, Sarah K. Wootton, Darwyn Kobasa

**Affiliations:** 1 Special Pathogens Program, National Microbiology Laboratory, Public Health Agency of Canada, 1015 Arlington Street, Winnipeg, MB R3E 3R2, Canada; 2 National Centre for Foreign Animal Disease, Canadian Food Inspection Agency, 1015 Arlington Street, Winnipeg, MB R3E 3M4, Canada; 3 Molecular Pathobiology, National Microbiology Laboratory NML, Public Health Agency of Canada, Winnipeg, MB R3E 3R2, Canada; 4 Department of Medical Microbiology and Infectious Diseases, Faculty of Health Sciences, College of Medicine, University of Manitoba, 745 Bannatyne Avenue, Winnipeg, MB R3E 0J9, Canada; 5 Department of Pathobiology, University of Guelph, Guelph, ON N1G 2W1, Canada

**Keywords:** SARS-CoV-2, COVID-19, pandemic, angiotensin-converting enzyme 2 (ACE2), virology, infection, host specificity, species specificity, adeno-associated virus, animal model, mouse model

## Abstract

Severe acute respiratory syndrome coronavirus 2 (SARS-CoV-2) is the aetiological agent of coronavirus disease 2019 (COVID-19) that has caused a pandemic with millions of human infections. There continues to be a pressing need to develop potential therapies and vaccines to inhibit SARS-CoV-2 infection to mitigate the ongoing pandemic. Epidemiological data from the current pandemic indicates that there may be sex-dependent differences in disease outcomes. To investigate these differences, we proposed to use common small animal species that are frequently used to model disease with viruses. However, common laboratory strains of mice are not readily infected by SARS-CoV-2 because of differences in the angiotensin-converting enzyme 2 (ACE2), the cellular receptor for the virus. To overcome this limitation, we transduced common laboratory accessible strains of mice of different sexes and age groups with a novel a triple AAV6 mutant, termed AAV6.2FF, encoding either human ACE2 or luciferase via intranasal administration to promote expression in the lung and nasal turbinates. Infection of AAV-hACE2-transduced mice with SARS-CoV-2 resulted in high viral titers in the lungs and nasal turbinates, establishment of an IgM and IgG antibody response, and modulation of lung and nasal turbinate cytokine profiles. There were insignificant differences in infection characteristics between age groups and sex-related differences; however, there were significant strain-related differences between BALB/c vs. C57BL/6 mice. We show that AAV-hACE2-transduced mice are a useful for determining immune responses and for potential evaluation of SARS-CoV-2 vaccines and antiviral therapies, and this study serves as a model for the utility of this approach to rapidly develop small-animal models for emerging viruses.

## 1. Introduction

In late 2019, a novel human coronavirus termed severe acute respiratory syndrome coronavirus 2 (SARS-CoV-2) emerged, likely due to spillover from an animal reservoir and is now causing a global pandemic [1]. As of July, 2022, there have been over 550 million confirmed infections worldwide, and over 6.3 million associated fatalities. SARS-CoV-2, the causative agent of coronavirus disease 2019 (COVID-19), is a single-stranded positive-sense RNA virus in the family Coronaviridae, which like SARS-CoV and Middle Eastern respiratory syndrome coronavirus (MERS-CoV), emerged due to spillover from a wildlife reservoir (bats) to the human population via an intermediate host(s) [2,3,4]. Although SARS-CoV-2 causes mild or asymptomatic disease in most cases, severe to critical illness occurs in a small proportion of infected people, with the highest rate seen in individuals 70 years of age or older and patients with comorbidities [5]. Given the magnitude of this public health emergency and the growing financial and mental health crisis caused by the COVID-19 pandemic [6], prophylactic and therapeutic measures that prevent transmission of the virus are urgently needed. Animal models that are susceptible to SARS-CoV-2 infection and recapitulate the disease are a critical for development of these essential medical countermeasures.

Several animal models are susceptible to infection with authentic SARS-CoV-2 including Syrian hamsters [7,8,9], deer mice [10], ferrets [11,12,13,14], and nonhuman primates (NHPs) [15,16,17,18,19]. These animal models support SARS-CoV-2 replication in the respiratory tract and other tissues, display quantifiable clinical signs including fever, weight loss, lethargy, etc., and develop mild-to-moderate disease [reviewed in [20]], thus they have been instrumental in the evaluation of vaccines and antiviral agents. However, these animal models are not without their limitations; there are limited research tools available for Syrian hamsters, particularly with respect to immunological analyses [20], ferrets are expensive, not as readily available, and require specialized housing [21], and NHPs are generally not an appropriate pre-clinical model due to ethical considerations and as well as considerable cost and limited supply. However, NHP are more often used as the final criterion for establishing the potential efficacy of a vaccine or therapeutic before transition to human clinical trials [22].

Mice on the other hand are highly versatile, affordable and well-studied laboratory model; however, they cannot be infected with most strains of SARS-CoV-2 because the murine ortholog of angiotensin-converting enzyme 2 (ACE2), the cell surface receptor for SARS-CoV-2, does not function as a receptor to initiate virus infection [23]. Fortunately, SARS-CoV-2 can infect mice genetically engineered to express human ACE2 [24,25,26,27], suggesting a lack of post-entry restrictions in this species. Nevertheless, these transgenic mice were initially available in limited quantities, and in some cases, permit replication of SARS-CoV-2 in the brain leading to severe neurological complications (encephalitis) [24,28,29,30], which few COVID-19 patients develop [31]. A mouse-adapted version of SARS-CoV-2 (SARS-CoV-2 MA) has been generated; however, the mutations that confer replication in mice are located in the receptor binding domain (RBD) of the spike protein, which might diminish the efficacy of some antibody therapies or vaccines in mice [32].

An alternative approach that has been used to sensitize mice to MERS-CoV [33], and more recently SARS-CoV-2 [34,35,36,37] infection is to use a viral vector to deliver the appropriate receptor gene to mouse lungs prior to infection with authentic patient-derived viruses. Here, we used the lung tropic AAV6.2FF vector [38] to deliver the human ACE2 (hACE2) gene to the upper and lower respiratory tract rendering mice of different genetic backgrounds permissive to SARS-CoV-2 infection. We selected this triple mutant AAV6 capsid containing F129L, Y445F, and Y731F mutations due to its high transducing efficiency in the upper and lower respiratory tract, including the nasal cavity, and for its ability to mediate rapid transgene expression [38]. This model can be generated quickly (2–3 weeks), is compatible with any strain of mouse without additional breeding, including knockout mice, and can be used for testing prophylactic and therapeutic strategies to combat COVID-19. We selected Balb/C mice as a common strain, that are widely used in pathogenesis and therapeutic evaluation studies, to assess the efficacy of AAV6.2FF mediated transduction with foreign genes. We also included selected groups of C57/BL6 mice to demonstrate the broader applicability of the AAV6.2FF mediated transduction to other mouse strains and for comparison of outcome following SARS-CoV-2 infection in a second common mouse strain. Given the plethora of available reagents, it is well suited for conducting detailed mechanistic, immunological and pathological analyses. Moreover, AAV6.2FF-vectored delivery of hACE2 allows for localized expression of hACE2 in the respiratory tract, or if delivered systemically, expression in other major organs, including the heart [39]. One advantage AAV has over Ad-vectored approaches, is AAV can mediate production of a transgene ensuing over 200 days [39], while in Ad-vectored approaches, transient gene expression usually is restricted to about 14 days [40]. This approach can be applied for the rapid development of relevant murine and other animal models for newly emerging viral infections.

## 2. Materials and Methods

### 2.1. Ethics Statement

The experiments described in this study were carried out at the National Microbiology Laboratory (NML) at the Public Health Agency of Canada as described in the Animal use document AUD# H-20-006. Experiments were approved by the Animal Care Committee located at the Canadian Science Center for Human and Animal Health in accordance with the guidelines provided by the Canadian Council on Animal Care. All procedures were performed under anesthesia, and all efforts were made to minimize animal suffering and to reduce the number of animals used. All infectious work with SARS-CoV-2 was performed under biosafety level 4 (BSL-4) conditions.

### 2.2. Cells and Viruses

Vero E6 (CRL-1586; American Type Culture Collection, Manassas, VA, USA) and Vero (CCL-81; American Type Culture Collection) were cultured at 37 °C + 5% CO_2_ in Dulbecco’s modified Eagle’s medium (HyClone, GE Healthcare Life Sciences, Logan, UT, USA) supplemented with 10% fetal bovine serum (FBS), 1% L-glutamine, and 100 U mL^−1^ penicillin–streptomycin.

The SARS-CoV-2 strain used in these studies (SARS-CoV-2; hCoV- 19/Canada/ON-VIDO492 01/2020, GISAID accession# EPI_ISL_425177) was isolated from a clinical specimen obtained at the Sunnybrook Research Institute (SRI)/University of Toronto on VeroE6 cells and provided by the Vaccine and Infectious Disease Organization (VIDO) with permission. Infectious stocks were grown by inoculating Vero CCL-81 cells and collecting supernatant upon observation of cytopathic effect; debris were removed by centrifugation at 6000× *g* for 5 min and stored at −80 °C until thawed.

To generate AAV6.2FF-hACE2, the human ACE2 gene (HG10108-M, Sino Biological Inc., Beijing, China) was cloned into an AAV2 genome plasmid downstream of the composite CASI promoter [41] followed by a woodchuck hepatitis virus posttranscriptional regulatory element (WPRE), a simian virus 40 polyadenylation sequence and flanked by AAV2 inverted terminal repeats (ITRs) as described previously [42]. AAV expressing firefly luciferase (AAV-Luc) was described previously [39]. AAV vectors were produced by two plasmid transfection of human embryonic kidney 293 cells (HEK293; CRL-1573, ATCC, Manassas, VA, USA) and heparin affinity column chromatography as described previously [43]. AAV vectors were titered by TaqMan quantitative polymerase chain reaction (qPCR) analysis as described [44,45]. 

### 2.3. Mouse Experiments

BALB/c and C57BL/6 were purchased from Jackson Laboratories (Bar Harbor, ME, USA). Animals were housed in groups and fed standard chow diets with food and water provided ad libitum. Mice of different ages and sexes (eight to ten weeks old to over 1 year old) were administered 1 × 10^11^ vector genomes (vg) of AAV6.2FF-hACE2 or AAV6.2FF-Luciferase via a modified-intranasal administration [46]. Individual groups consisted of young male Balb/C mice, young female Balb/C, old male Balb/C mice, old female Balb/C mice, young male C57BL/6 mice and young female C57BL/6 mice. Each group had 36 mice equally divided between male and female with 18 mice transduced with AAV6.2FF-hACE2 and 18 mice transduced with AAV6.2FF-Luciferase. In brief, mice were anesthetized with isoflurane and the viral vector administered via the intranasal route (i.n) in a volume of 50 μL. The mouth was covered during administration to promote inhalation through the nose and subsequent distribution of the vector throughout the lower respiratory tract. Ten days after AAV transduction, mice were inoculated with 10^5^ TCID50 of SARS-CoV-2 in a 50 μL volume given i.n. Weights were monitored on a daily basis, and animals were sacrificed at 2, 4, or 28 dpi (*n* = 6 mice (3 male + 3 female) per time point) and tissues were harvested. Mice were euthanized under isoflurane anesthesia after serum collection via cardiac puncture and cervical dislocation before tissue collection.

### 2.4. Infectious Virus Quantification

For infectious virus assays, 50% tissue culture infective dose (TCID50) was calculated using the Reed and Muench method [47] and expressed as TCID50 per gram of sample (TCID50/g). Briefly, harvested tissue samples for infectious assays were flash frozen and stored at −80 °C. Thawed tissue samples were weighed and placed in 1 mL of minimal essential medium (MEM, HyClone, GE Healthcare Life Sciences, Logan, UT, USA) supplemented with 1% FBS (Gibco, Life Technologies, Grand Island, NY, USA), 1× L-glutamine (Gibco, Life Technologies, Grand Island, NY, USA), and 100 U mL^−1^ penicillin–streptomycin (Gibco, Life Technologies, Grand Island, NY, USA) before being homogenized in a Bead Ruptor Elite Bead Mill Homogenizer (Omni International, Kennesaw, GA, USA) at 4 m/s for 30 s. Lysates were clarified by centrifugation at 1500× *g* for 6 min. Tissue homogenates were serially diluted in MEM supplemented with 1% heat-inactivated FBS, 1× L-glutamine, and 200 U mL^−1^ penicillin–streptomycin. One hundred microliter volumes of sample dilutions were added to 96-well plates of 95% confluent Vero cells containing 50 μL of the same medium in three replicates and incubated for 5 days at 37 °C with 5% CO_2_. Plates were monitored daily and scored for the presence of cytopathic effect on day 3–5 after infection.

### 2.5. Reverse Transcriptase-Quantitative Polymerase Chain Reaction

Tissue samples harvested for viral RNA (vRNA) detection were immersed in RNAlater (Ambion, Austin, TX, USA) 4 °C for 1 day, then stored at −80 °C until later use. Tissue samples were thawed and weighed and homogenized in 600 μL RLT buffer using a Bead Ruptor Elite Bead Mill Homogenizer (Omni International, Kennesaw, GA, USA) with a stainless steel bead at 4 m/s for 30 s. Viral RNA from 30 mg tissue samples was extracted with the RNeasy Plus Mini kit (Qiagen). For detection of SARS-CoV-2 RNA, SARS-CoV-2 E Sarbeco real-time RT–PCR assay [48], recommended by the WHO was used. The primers used were E_Sarbeco_F1 (5′-ACAGGTACGTTAATAGTTAATAGCGT-3′) and E_Sarbeco_R2 (5′-554 ATATTGCAGCAGTACGCACACA-3′). The probe was E_Sarbeco_P1 (5′-FAM555 ACACTAGCCATCCTTACTGCGCTTCG-BBQ-3′). The assay was set up using the TaqPath 1-Step Multiplex Master Mix kit (Applied Biosystems, Thermo Fisher Scientific, Waltham, MA, USA) on a QuantStudio 5 real-time PCR system (Applied Biosystems, Thermo Fisher Scientific, Waltham, MA, USA), as per manufacturer’s instructions. A standard curve was generated by using plasmids coding for the SARS-CoV-2 E gene and were used on each plate for the quantification of copy numbers.

### 2.6. Serum ELISA

SARS-CoV-2 spike specific IgM and IgG antibody responses were determined using an in-house enzyme-linked immunosorbent assay (ELISA). Briefly, 96 well plates (Corning Inc, Corning, NY, USA) were pre-coated with full length spike or EBOV GP (negative control) overnight and blocked with phosphate-buffered saline (PBS) containing 5% skim milk for one hour. A serial dilution of mouse serum was carried out in triplicate in PBS containing 5% skim milk before being applied to the plate, incubated for one hour, and then washed three times in PBS plus 0.1% Tween 20 using a BioTek plate washer. Mouse IgG was detected with a Goat anti-Mouse IgG (H + L) Secondary Antibody conjugated with HRP (Catalog # 31430; 1:10,000 dilution, Invitrogen, Thermo Fisher Scientific, Waltham, MA, USA). Mouse IgM was detected with a Goat anti-Mouse IgM (H + L) Secondary Antibody conjugated with HRP (Catalog # 31440; 1:10,000 dilution, Invitrogen, Thermo Fisher Scientific, Waltham, MA, USA). Both IgG and IgM secondary antibodies were incubated for one hour Following a second set of washes, the plate was incubated with 3,3′,5,5′-tetramethylbenzidine (TMB) Single Solution substrate (Thermo Fisher Scientific, Waltham, MA, USA). The reaction was stopped with 1N sulfuric acid and the absorbance was read at 450 nm using a BioTek Synergy HT plate reader (BioTek, Winooski, VT, USA).

### 2.7. Cytokine Analyses

The murine cytokine response was quantified by a custom ordered TaqMan™ Gene Expression Assay (FAM) (Applied Biosystems, Thermo Fisher Scientific, Waltham, MA, USA). The following cytokines were analysed: IL-1 alpha (Mm00439620_m1), IL-1 beta (Mm00434228_m1), IFN alpha (Mm03030145_gH), IFN beta (Mm00439552_s1), IFN gamma (Mm00434256_m1), granzyme A (Mm00439191_m1), granzyme B (Mm00442834_m1), TNF-alpha (Mm00434256_m1), TGF-beta (Mm01227699_m1), IL-2 (Mm00434256_m1), IL-4 (Mm00434256_m1), IL-6 (Mm00434256_m1), IL-8 (Mm04208136_m1), IL-10 (Mm00434256_m1), IL-12p40 (Mm01288993_m1), IL-17 (Mm00439618_m1), MCP1 (Mm99999056_m1), VEGF (Mm00437306_m1), and GAPDH (Mm99999915_g1) as a housekeeping gene control. RNA was extracted from tissues using the RNeasy plus mini kit (Qiagen, Hilden, Germany) following the manufacturer’s instructions. For removal of genomic DNA and reverse transcription, SuperScript IV VILO Master Mix with ezDNase Enzyme (Invitrogen, Thermo Fisher Scientific, Waltham, MA, USA) was used as per manufacturers protocols. TaqMan^®^Fast Universal PCR MasterMix (2×) (Applied Biosystems, Thermo Fisher Scientific, Waltham, MA, USA) was used according to the manufacturer’s instructions in a two-step qRT-PCR reaction in triplicate. The PCR was performed on a Quantstudio 5 (Applied Biosystems, Thermo Fisher Scientific, Waltham, MA, USA). One microliter (10 ng) of cDNA sample was assayed per reaction. Each reaction consisted of 1 cycle of 95 °C for 20 s, followed by 50 cycles of 95 °C for 3 s and 60 °C for 30 s. The ΔΔCt method was used with normalization within a sample on GAPDH (ΔCt) calculated for each gene. The comparison was against hACE2-transduced mice vs. Luciferase transduced mice (ΔΔCt) or Luciferase transduced mice vs. mock-uninfected mice (ΔΔCt). 

### 2.8. Western Blot 

HEK 293 cells were transfected with pACASI-hACE2-WPRE or pACASI-Luc-WPRE using PEI MAX™ (Polysciences, Inc., Warrington, PA, USA) as per manufacturer’s instructions. 48 h post-transfection, cells were washed twice with cold PBS, collected and pelleted at 450 × g 5 min. The cell pellet was lysed in 500 μL cold RIPA buffer (50 mM Tris HCl, pH 7.5; 150 mM NaCl; 1% Triton X-100; 0.1% SDS; 10 mM EDTA; 1% sodium deoxycholate) supplemented with protease inhibitor cocktail (Roche Applied Science, Penzberg, Germany) and incubated on ice for 30 min. Samples were centrifuged at 18,000× *g* for 15 min at 4 °C to remove cell debris. Twenty microlitres of cell lysate was separated on a 12% SDS PAGE gel, transferred to polyvinylidene fluoride (PVDF) membrane and blocked overnight at 4 °C in blocking solution (5% skimmed milk and 0.1% Tween 20 in PBS). The membrane was incubated 1 h at RT with a goat anti-ACE2 antibody (Cat no. AF933 R&D Systems, Minneapolis, MN, USA) diluted 1:500 in blocking solution. Membranes were washed five times in washing buffer (0.1% Tween 20-PBS) for five minutes and then incubated for 1 h at RT with an HRP conjugated donkey anti-goat IgG secondary antibody (Cat. No. PA1-28664, Thermo Fisher Scientific, Waltham, MA, USA) diluted 1:5000 in blocking solution. The membrane was washed five times with washing buffer and the chemiluminescent signal was detected using SuperSignal West Pico PLUS chemiluminescent substrate (Thermo Fisher Scientific, Waltham, MA, USA) and imaged with a ChemiDocTM Molecular Imager (Bio-Rad, Hercules, CA, USA). Molecular Weight (MW): GeneDireX BLUelf Prestained Protein Ladder Cat. PM008-0500 (GeneDireX Inc, Taiwan, China).

### 2.9. Immunofluorescence Analysis of hACE2 Expression in Murine Lungs

Groups of four mice transduced with 1 × 10^11^ vg of AAV6.2FF-hACE2 or AAV6.2FF-Luciferase intranasally were euthanized 10 days post-vector administration and exsanguinated via heart puncture. Lungs were perfused via the heart with PBS and then instilled with optimal cutting temperature compound (OCT)/PBS (1:1) via the trachea. Lungs were removed en bloc and placed directly into an OCT containing cryomold and placed on dry ice to freeze. Solid blocks of lung tissue were stored at −80 °C. 8 μm cryosections were placed on Superfrost Plus microscope slides (Thermo Fisher Scientific, Waltham, MA, USA) and allowed to dry at RT for 3 h. Slides were fixed in ice-cold acetone for 15 min and then washed twice in 0.1% Tween 20-PBS for 5 min each followed by a 5 min wash in PBS. Antigen retrieval was performed by heating antigen retrieval buffer (10 mM sodium citrate, 0.05% Tween-20, pH 6.0) to 100 °C in a microwave and placing slides in heated solution for 10 min for a total of three times. Slides were then washed in PBS for 5 min and a hydrophobic circle was drawn around the lung tissue using a Dako pen. Lung tissue was permeabilized in 0.1% Triton X-100-PBS for 10 min at RT and blocked with 10% FBS-PBS for 1 h at RT. Rabbit anti-ACE2 mAb (Cat no. ab108209 Abcam Inc., Toronto, ON, Canada) diluted 1:100 in the blocking buffer was added to slides and incubated at 4 °C for two days. Slides were washed three times with PBS for 10 min each before being incubated with goat anti-rabbit Alexa 488 (Cat. no. A-11034, Life technologies, Carlsbad, CA, USA) diluted 1:500 in blocking buffer for 1 h at RT. Slides were washed three times with PBS for 10 min each and mounted using ProLong Diamond Antifade Mountant with DAPI (Thermo Fisher Scientific, Waltham, MA, USA). Slides were imaged using an Axio Observer inverted fluorescence microscope (Carl Zeiss MicroImaging GmbH, Göttingen, Germany). 

### 2.10. In Vivo Luciferase Imaging

Mice that were administered a total of 1 × 10^11^ vector genomes of AAV6.2FF-Luc or AAV6.2FF-hACE2 intranasally (Section 2.3) were used for detection of luciferase expression. Bioluminescence imaging was performed on days 3, 5, 7, and 10 after intraperitoneal (IP) administration of XenoLight RediJect D-Luciferin (Perkin Elmer, Waltham, MA, USA) at a concentration of 150 mg per kg using the IVIS SpectrumCT instrument (Perkin Elmer, Waltham, MA, USA). Resultant data were analysed and the signal intensity quantified using Living Image software (Perkin Elmer, Waltham, MA, USA).

### 2.11. Histology and Immunohistochemistry

Tissues were fixed in 10% neutral phosphate buffered formalin, routinely processed, sectioned at 5 μm and stained with hematoxylin and eosin (HE) for histopathologic examination. Paraffin tissue sections were quenched for 10 min in aqueous 3% hydrogen peroxide. Epitope retrieval was performed using an in-house glycan retrieval solution in a Biocare Medical Decloaking Chamber (Biocare Medical, Pacheco, CA, USA). The primary antibody applied to the sections was SARS-CoV-2 (2019-nCoV) Nucleocapsid, Rabbit MAb (#40143-R019, Sino Biological Inc., Beijing, China) used at a 1:6000 dilution for thirty minutes. They were then visualized using a horse radish peroxidase labelled polymer, Envision^®^ + system (anti-rabbit) (Dako, Santa Clara, CA, USA) and reacted with the chromogen diaminobenzidine (DAB). The sections were then counter stained with Gill’s hematoxylin. Semi-quantitative lesion scoring was performed as follows: The percentage affected of each section examined was scored as 0 = no pathological changes, 1 = ≤25% of lung section affected, 2 = >25% and ≤50% of lung section affected, 3 = >50% and ≤75% of lung section affected and 4 = >75% of lung section affected. Additionally, each section was assigned a severity score or 0 = no lesions, 1 = mild lesions, 2 = moderate lesions and 3 = severe lesions Additionally, a score of 0 for not present and 1 for present was given for each of the following parameters and an average score out of 8 was assigned for histological features present: bronchiolitis (including inter-epithelial inflammatory cells, necrosis of bronchiolar epithelium and debris in lumen), diffuse alveolar damage (including necrosis of alveolar epithelial cells, cellular debris in alveoli and intra-alveolar fibrin), alveolar edema, alveolar hemorrhage, hyperplasia of type II pneumocytes, perivasculitis and vasculitis and presence of multinucleated cells. In total, a score was assigned for each section which included percentage affected (/4), severity (/3) and histological features (/8) for a total score /15. For most groups, in which 4 sections were examined the scores from all the sections in each group were added together and are presented in Table S1. In one group in which 8 sections were examined the total score was divided by 2.

### 2.12. Data Analysis 

Results were analysed and graphed using Prism 8.2.1 software (Graphpad Software, La Jolla, CA, USA). The statistical significance between the groups was determined using a Mann–Whitney test or one-way and two-way analysis of variance (ANOVA).

## 3. Results

### 3.1. Generation of an AAV6.2FF-hACE2 and AAV6.2FF-Luciferase

**Figure 1:**
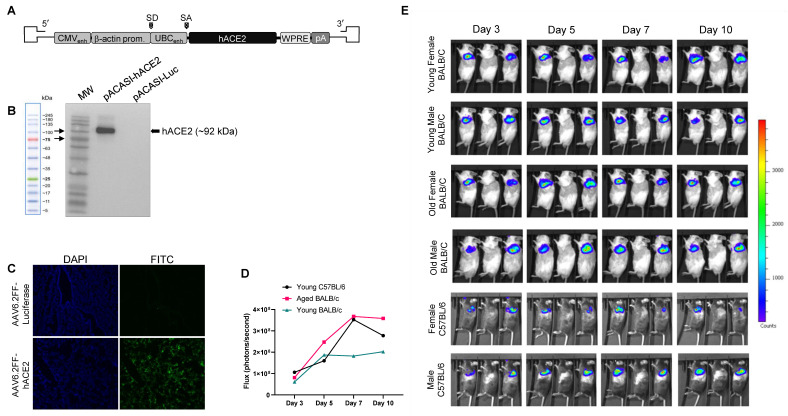
(**A**) Schematic of the AAV genome expressing human ACE2 (hACE2) under the control of the composite CASI promoter (containing cytomegalovirus immediate early promoter (CMV), chimeric chicken-β-actin (CAG), and ubiquitin C (UBC) enhancer region) and flanked by a Woodchuck Hepatitis Virus Posttranscriptional Regulatory Element (WPRE) and an SV40 polyA sequence (pA) as well as splice donor (SD) and acceptor (SA) sequences. (**B**) HEK 293 cells transfected with pACASI-hACE2-WPRE or pACASI-Luc-WPRE (**C**) Immunofluorescence staining of mouse lung sections demonstrating widespread hACE2 expression in AAV6.2FF-hACE2-transduced mice at 10 days post-vector administration. (**D**) Quantification of luciferase expression in AAV-Luciferase-transduced mice. (**E**) IVIS imaging of AAV-Luc (right and left) transduced mice and AAV-hACE2-transduced mice (middle). Total mice per transduced group (Luc or hACE2) is 6 (3 male and 3 female) with representative imaging shown for 3 mice per day per group.

To develop the AAV6.2FF vectors for expressing hACE2 and luciferase in mouse lungs and nasal turbinates, hACE2 and firefly luciferase cDNA were cloned into an AAV genome containing the CASI promoter, a WPRE, and an SV40 polyA signal located between AAV2 inverted terminal repeats (Figure 1A). hACE2 expression was validated by transducing HEK 293 cells and analysing cell lysates for hACE2 expression using a Western blot (Figure 1B). To confirm hACE2 expression in lungs of transduced mice, immunofluorescence analysis (IFA) was performed. Mice transduced with 1 × 10^11^ vg of AAV6.2FF-hACE2 or AAV6.2FF-Luciferase intranasally were euthanized 10 days post-vector administration and lungs were harvested after perfusion via the heart. Potent expression of hACE2 was observed (Figure 1C). To determine the optimal day post vector administration for infection experiments, we administered of 1 × 10^11^ vg of AAV6.2FF expressing CASI-driven firefly luciferase (AAV-Luc) reporter gene which demonstrated potent transduction in the upper and lower lungs. The transduction of foreign genes from the AAV6.2FF vector was modelled by measuring luciferase expression, which can be directly visualized at sequential time points. Luciferase expression was then used to infer the kinetics of expression of hACE2 in mice. The flux (in photons/second) generated from the IVIS scanner was used to determine the intensity of luciferase expression and was captured on days 3, 5, 7, and 10 post AAV vector administration. As early as 3 days after administration, luciferase expression was seen (Figure 1D,E). Differences were observed in luciferase expression between female and male mice. The luciferase signal continued to increase until peak luciferase expression was seen in young female BALB/c, old female BALB/c, and female C57BL/6 mice at 7 dpi. Luciferase expression in young male BALB/c, old male BALB/c, and male C57BL/6 mice continued to increase up to day 10, the last time point tested. We also observed greater luciferase expression in female mice than male mice within the same age group and strain. Luciferase expression was the greatest on day 10 overall, although we did not look at times after day 10. We considered that expression was sufficient by day 10 and selected day 10 post vector administration for SARS-CoV-2 infection experiments.

### 3.2. AAV6.2FF-hACE2-Transduced Mice Are Permissive to SARS-CoV-2 Infection

**Figure 2:**
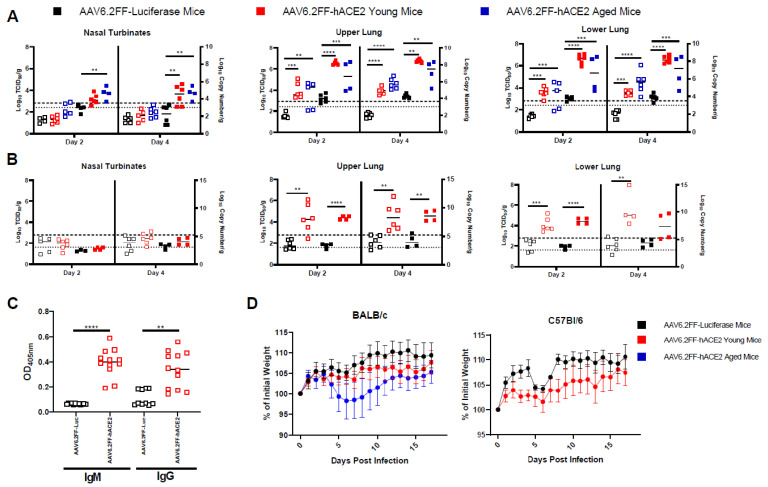
SARS-CoV-2 viral titers, weight loss, and antibody response in AAV transduced, infected mice. Infectious viral titers (open squares; left Y axis; dashed line is limit of detection (LOD)) or viral RNA copy numbers (filled squares; right Y axis; dotted line LOD) were detected in the nasal turbinates, upper lung, and lower lung of infected AAV6.2FF-Luciferase transduced mice (black symbols), or young AAV6.2FF-hACE2-transduced mice (red symbols), or aged AAV6.2FF-hACE2-transduced mice (blue symbols) in 6 mice (3 male, 3 female) per time point per group. (**A**) shows data for BALB/c mice, while (**B**) shows data for C57Bl/6 mice. (**C**) Anti-SARS-CoV-2 spike antibody responses in infected, AAV6.2FF-hACE2-transduced BALB/c and C57Bl/6 mice on day 28 post infection compared with luciferase transduced mice. (**D**) Weight loss seen in both strains of mice throughout the course of infection. Statistical significance was assessed by ANOVA (**A**,**D**, BALB/c), or unpaired T test (**B**–**D**, C57/Bl6). ** = *p* < 0.01, *** = *p* < 0.001, **** = *p* < 0.0001.

Here, 8–10 week (young) female/male BALB/c, 1-year (old) female/male BALB/c, and 8–10 week (young) female/male C57BL/6 mice were administered 1 × 10^11^ vg of AVV6.2FF expressing hACE2 or luciferase (control). The mice were then inoculated with 10^5^ TCID50 of SARS-CoV-2 by an intranasal route (i.n.) 10 days post vector administration and monitored daily for clinical signs and weight loss for 28 days. Mice were sacrificed at 2, 4, and 28 days post-infection (dpi) with 3 male mice and 3 female mice per time point and group. SARS-CoV-2 inoculated mice did not succumb to infection and no significant weight loss was observed (Figure 2D). Clinical signs of infection were only apparent in hACE2-transduced old male BALB/c mice, which displayed ruffled fur and rapid respiratory rate only at 8 and 9 dpi. At 2 and 4 dpi, the hACE2-transduced mice had high levels of virus and viral RNA in the lungs (Figure 2A,B). RNA copy values ranged from 5.1–9.5 Log10 copies/g at 2 dpi to 5.4–9.7 Log10 copies/g at 4 dpi and infectious virus ranging from 2.1–5.5 Log10 TCID50/g at 2 dpi to 2.2–5.7 Log10 TCID50/g at 4 dpi were detected in the upper and lower lung, respectively. Viral RNA was also present in the nasal turbinates, however most samples did not have infectious viral loads above the limit of detection (Figure 2A,B). The AAV-Luc transduced mice showed low levels of viral RNA (ranging from 3.4–5.1 Log10 copies/g at 2 dpi to 3.2–4.9 Log10 copies/g at 4 dpi), but the majority of animals had infectious titers below the limit of detection. Upper and lower lung titres were very similar in the AAV-hACE2 group, with nasal turbinate titers being low, likely due to the transduction efficiency of AAV-hACE2 in these locations. In both strains of AAV-hACE2-transduced mice, viral RNA and infectious virus in the lungs and nasal turbinates were very similar at 2 dpi and 4 dpi indicating sustained replication of virus over these time points. Age and sex (Figures S1 and S2) generally did not make a significant contribution to the viral burden detected in the tissues of BALB/c mice. Surprisingly, old male hACE2-BALB/c, which showed a few clinical signs, did not have significantly higher lung titres than young male hACE2-BALB/c mice. In C57BL/6 mice there was a significant difference in lung titres in male and female groups suggesting differential strain specific responses to either AAV or SARS-CoV-2, however there were only two animals of each sex examined here, and any differences will have to be examined further.

Serum samples collected from mice were analysed by indirect IgG and IgM ELISA using full length SARS-CoV-2 spike as antigen. At day 28, AAV-hACE2-transduced mice challenged with SARS-CoV-2 developed IgG and IgM antibodies against SARS-CoV-2 spike protein. By contrast, antibody responses in the SARS-CoV-2 challenged AAV-Luc group were significantly weaker. In these animals IgM was undetectable and IgG response was weak or not detected (Figure 2C).

### 3.3. Histopathology and Immunohistochemistry

**Figure 3:**
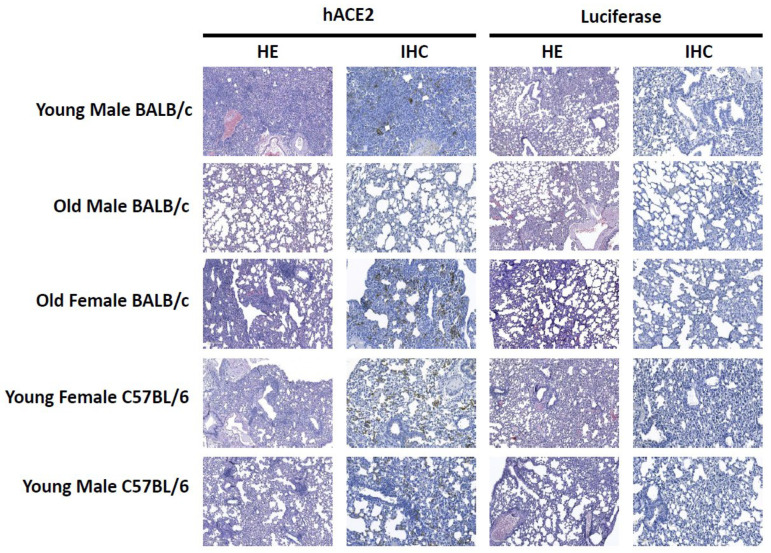
Presence of microscopic lesions (HE, Hematoxylin/eosin stain) and antigen distribution by immunohistochemistry (IHC) in tissues of SARS-CoV-2 infected mice. Mice of different sex and strain were transduced with either hACE2-AAV6.2FF or Luciferase-AAV6.2FF and inoculated with SARS-CoV-2. The magnification is 10× for H&E and IHC. Scale bars = 100 um.

Upon necropsy, no gross lesions or hemorrhages were observed in the lungs for any group. We further assessed the impact of SARS-CoV-2 infection on lung histopathology by hematoxylin and eosin (H&E) and immunohistochemistry (IHC). The H&E and IHC scores are summarized in Table S1. The lesions observed by H&E were variable and ranged in severity between individual animals. On average, at 2 dpi there is a slightly higher pathology score in the AAV-hACE2-transduced mice compared to the AAV-Luc transduced mice in all groups except for in the young male C57Bl/6 group at 2 dpi. At 4 dpi there is a higher pathology score for the AAV-hACE2 mice for all groups except for the old male BALB/c where the AAV-Luc-transduced animals have a higher score. Histology and immunohistochemistry findings at 4 dpi are presented in Figure 3. In the young male BALB/C group the AAV-hACE2-transduced mice show severe lesions including interstitial infiltrates of inflammatory cells, necrosis, type II pneumocyte hyperplasia, vasculitis and perivasculitis. In contrast, the AAV-Luc transduced mice show only multifocal interstitial infiltrates of inflammatory cells. Surprisingly, in the old male BALB/c group, more severe lesions were observed in the AAV-Luc mice and milder lesions in the AAV-hACE2-transduced group at 4 dpi. In the old female BALB/C group, similar findings including interstitial infiltrates of inflammatory cells, perivasculitis, and type II pneumocyte hyperplasia were observed in both the AAV-hACE2-transduced and AAV-Luc mice. However, the lesions were more severe in the AAV-hACE2-transduced mice and additional lesions of vasculitis and subendothelial accumulation of inflammatory cells were only observed in the AAV-hACE2-transduced mice. In the young male C57BL/6 group similar lesions that included prominent perivascular inflammation, hemorrhage and interstitial infiltrates of inflammatory cells were observed in both the AAV-hACE2-transduced and AAV-Luc mice; however, lesions were slightly more severe in the AAV-hACE 2-transduced mice at 2 dpi. In the young female C57BL/6 group both the AAV-hACE2-transduced and AAV-Luc mice showed lung inflammation with a predominantly perivascular distribution; however, the AAV-hACE2 mice showed more severe lesions with additional features of necrosis, vasculitis and type II pneumocyte hyperplasia. There was no antigen detected in any of the AAV-Luc-transduced groups. Antigen was detected in all of the AAV-hACE2-transduced groups except the old male BALB/C.

### 3.4. SARS-2-CoV Infection Induces a Modulation of Immune Responses in hACE2-Transduced Mice

The difference in the SARS-CoV-2 challenge of the different mouse groups prompted us to explore the host cytokine/chemokine responses in the lung and nasal turbinates. Cytokine expression was quantified in the upper lung, lower lung, and nasal turbinates by real-time quantitative PCR (qRT-PCR) using a panel that includes IL-1α, IL-1β, IFN-α, IFN-β, IFN-γ, granzyme A, granzyme B, TNF-α, TGF-β, IL-2, IL-4, IL-6, IL-8, IL-10, IL-12p40, IL-17, MCP1, VEGF, and GAPDH as a housekeeping control. Overall, viral titres were greatest at 4 dpi, hence we chose 4 dpi to measure the cytokine responses. Initially, we evaluated the impact of SARS-CoV-2 infection in AAV-Luc transduced/SARS-CoV-2-innoculated mice only (Figure S4) and compared relative expression levels to age, sex, and strain matched uninfected mice by ΔΔCt method. This was done to determine whether AAV transduction without productive SARS-CoV-2 replication led to an increase in transcription of cytokine genes. Among all young mice, there were no significant differences in cytokine expression in the upper lung, lower lung, or nasal turbinates in response to AAV-Luc transduction and SARS-CoV-2 delivery. Several genes were weakly modulated in the SARS-CoV-2 infected AAV-Luc old male BALB/c mice with differing degrees of expression. Tissue specific modulation of gene expression was apparent for IL-1β and granzyme A for the old male mice. 

**Figure 4:**
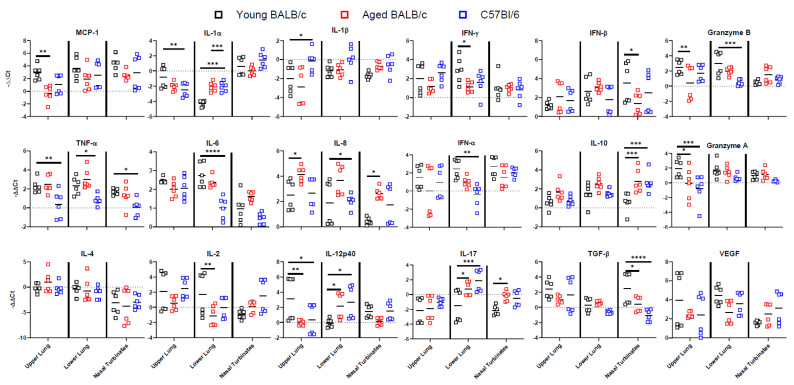
Cytokine gene expression in SARS-CoV-2 infected mice. Gene expression of selected cytokines normalized to GAPDH (ΔΔCt values) and compared to luciferase transduced mice in the upper lung, lower lung, and nasal turbinates on day 4 post-SARS-CoV-2 infection. All mice shown here were mice transduced with AAV6.2FF-hACE2 prior to SARS-CoV-2 infection. Comparisons between young and aged BALB/c mice and BALB/c and C57Bl/6 mice were done using two-way analysis of variance. * = *p* < 0.05, ** = *p* < 0.01, *** = *p* < 0.001, **** = *p* < 0.0001. *n* = 6/group TNF-alpha, Tumour Necrosis Factor alpha; TGF-beta, Transforming growth factor beta; MCP1, Monocyte Chemoattractant Protein-1; VEGF, vascular endothelial growth factor; GAPDH, Glyceraldehyde 3-phosphate dehydrogenase.

Next, we compared the cytokine expression of SARS-CoV-2 infected AAV-hACE2-transduced mice to AAV-Luc transduced mice (ΔΔCt) (Figure 4 and Figure S3). There were significant differences between AAV-hACE2 and AAV-Luc mice following infection with SARS-CoV-2. There were increases in gene expression found in IFN alpha, IFN beta, IFN gamma, granzyme A, granzyme B, TNF-α, TGF-β, IL-6, IL-8, IL-10, MCP1, and VEGF in AAV6.2FF-hACE2-transduced mice. There was a significant decrease in IL-1α, IL-1β, and IL-4 in AAV6.2FF-hACE2-transduced mice. There were some notable differences in genes that were associated with pro-inflammatory and antiviral responses that were tissue and strain specific. For example, the proinflammatory cytokine IL-17 was downregulated in the upper lung and nasal turbinates; however, in the lower lung, gene expression was downregulated only for young BALB/c mice and there was upregulation in old female BALB/c and male/female C57BL/6 mice. IL-1 alpha expression was downregulated only for the lungs but slightly upregulated in the nasal turbinates in all groups. There was a large variance in expression between the strains of mice, but low variation between the three mouse replicates of each sex in each mouse group. There was no clear trend seen with regard to differences between the age, sex, or strain of mice. Taken together, these results indicate that AAV-hACE2-transduced mice respond to SARS-CoV-2 infection by eliciting acute inflammatory responses and mice not expressing the hACE2 gene generally do not produce a cytokine response following infection with SARS-CoV-2.

## 4. Discussion

In this study, we have developed and characterized a mouse model of SARS-CoV-2 infection using AAV6.2FF-hACE2 mediated gene delivery to the mouse respiratory system and characterized the infection in BALB/c and C57BL/6J mice of different ages and sex. As expected, we confirmed that that AAV6.2FF mediated gene delivery was able to transduce gene expression in the lungs and nasal turbinates of mice, as previously demonstrated [37]. In both mouse species, early in the infection, mice transduced with AAV-hACE2 and infected with SARS-CoV-2 have high viral titres in respiratory tissues not seen in control AAV-Luc-transduced mice. AAV6.2FF mediated gene delivery of hACE2 was able to successfully generate a mouse model for SARS-CoV-2 infection that shows sites of infection that are comparable to those that are infected in species that are naturally susceptible to infection such as Syrian hamsters and deer mice [7,29]. This outcome is similar to the results of others that have similarly demonstrated AAV mediated transduction of the mouse airway with hACE2 using AAV9 and AAV2/8 [34,38,49]. Mice transduced with AAV-hACE2 produce both a cytokine and chemokine response in the lungs and nasal turbinates, whereas mice transduced with AAV-Luc did not produce a strong inflammatory response as a consequence of SARS-CoV-2 infection. Our results show that an IgG and IgM antibody response was generated in AAV-hACE2 mice after infection, while only a weak response was detected in AAV-Luc transduced mice. Thus, this animal would be a valuable model to improve our understanding of SARS-CoV-2 infection using a readily accessible small animal model. In addition, this model will allow for testing of vaccine and therapeutic candidates, as has been done by our group [42].

Although there are a few limitations of the AAV6.2FF system including large multiplicity of infection with AAV-hACE2, and potentially the cloning capacity of the AAV vector, development of AAV vectors expressing the hACE2 was efficient and rapid, resulting in the generation of a reproducible murine model for SARS-CoV-2 within three weeks. This short time course compares favorably with the much longer time required to develop transgenic mice expressing hACE2. Further, hACE2 expression after AAV-hACE2 intranasal inoculation is restricted to the respiratory system whereas transgene expression may be widespread [26,50,51] and result in infection in organs that are not usually associated with SARS-CoV-2 infection such as direct infection in brains of K18 hACE2 transgenic mice, which is a major factor contributing to the fatal outcome. Although AAV6.2FF-Luc transduced mice also had lesions and inflammation upon SARS-CoV-2 infection in the absence of SARS-CoV-2 antigen detection, inflammation associated with DMEM intranasal instillation of liquid into the lungs has been shown to cause instillation-associated histopathology [52]. Advantages that AAV vectors have over adenovirus-vectors (Ad) is that AAV is not strongly immunogenic, which was confirmed during the cytokine analysis in the mice given AAV-Luc, AAV-based transduction results in long lasting gene expression, and the tissue tropism of AAV can also be tailored using different serotypes.

We observed that the virus titers in the nasal turbinates and lungs, as well as manifestations of infection, were somewhat variable across the different groups of AAV-hACE2-transduced mice. One interesting group of mice were the older male BALB/c mice. These mice exhibited more apparent clinical signs such as rapid breathing and ruffled fur and considerably greater weight loss compared to other groups. Cytokine and chemokine responses were not dramatically different from the other groups of mice; however, the old male BALB/c transduced with AAV6.2FF-Luc did show a cytokine and chemokine response in 14/18 genes tested which suggests that these mice have some age-related innate immune system differences compared to the other groups of mice. Further to differences related to age, IHC results indicated that AAV6.2FF-hACE2-transduced old male mice did not have detectable viral protein antigens in contrast to the old female animals that had scores similar to the other mouse groups. In considering an explanation for the lack of antigen detection in older male mice by IHC despite detection of significant viral replication, we suggest that there may be differences in the distribution of replicating virus in older male animals compared to other groups. While the same lung lobes were consistently collected from each animal in each group for evaluation of virus titer or pathology, measurement of virus titers was done on a different lobe than that used for pathology. The differences in the older male animals might suggest differences in the sites of preferential virus replication in aged male mice compared to the other groups, and that virus replication was considerably limited in the lung lobe used for pathology. Further evaluation of the tissue specific distribution of viral replication in each group would be needed to confirm this possibility. However, the results do suggest possible aged and sex-related differences in outcome. Similar to infected patients, young AAV6.2FF-hACE2–transduced mice with normal immune systems developed mild disease whereas older male mice, like old male patients [53], were more profoundly affected, as weight loss and rapid breathing were observed in these mice. In COVID-19 patients with acute respiratory illness, the main clinical manifestation is severe lung inflammation. Future studies using other strains of mice may be needed to address the susceptibility of old male mice to infection with SARS-CoV-2 using this model.

Several infectious models have been characterized such as Syrian hamsters [7,8,9], ferrets [11,12,13,14], deer mice [10], and non-human primates [15,16,17,18,19]. These models are valuable tools for the study of SARS-CoV-2 infection and COVID-19 disease; however, the mouse’s small size, small housing requirements, and availability make the mouse model ideal for large scale studies without the lethal infection of the central nervous system (CNS) that is seen with the hACE2-transgenic mouse models. Continued development of this mouse model for SARS-CoV-2 will contribute to the development of vaccines, therapeutic agents and other countermeasures as a means to further enhance protection of the human population against infections by this pathogen.

## Data Availability

All data are available upon request, and inquiries should be sent to darwyn.kobasa@phac-aspc.gc.ca.

## Supplementary Materials

The following supporting information can be downloaded at: https://www.mdpi.com/article/10.3390/v15010085/s1: Figure S1: Comparison of viral titer, RNA levels, and weight loss in male and female BALB/c mice; Figure S2: Comparison of viral titer, RNA levels, and weight loss in male and female C57Bl/6 mice; Figure S3: Cytokine gene expression in SARS-CoV-2 infected mice; Figure S4: Cytokine gene expression in AAV6.2FF-Luciferase transduced mice compared to expression levels to age, sex, and species matched uninfected mice; Table S1: Hematoxylin/eosin (H&E) staining and in situ hybridization (ISH) scoring of SARS-CoV-2-infected lung tissue in mice at 4 dpi.
