# Supplementary material for: Generation and Characterization of a SARS-CoV-2-Susceptible Mouse Model Using Adeno-Associated Virus (AAV6.2FF)-Mediated Respiratory Delivery of the Human ACE2 Gene
Source: Viruses. 2022 Dec 28;15(1):85. doi: 10.3390/v15010085 (PMC9863330; doi:10.3390/v15010085)
Supplement: Supplementary file 1 [file viruses-15-00085-s001.zip › Table S1.pdf]

Table S1:

	Luc 2 DPI		hACE2 2 DPI		Luc 4 DPI		hACE2 4 DPI	
	HE	IHC	HE	IHC	HE	IHC	HE	IHC
Young Male Balb C	13	0	16	9	14	0	22	9
Old male BalbC	11	0	14	0	21	0	8	0
Old Female Balb C	16	0	20	11	15	0	20	8
Young Male C57Bl/6	15	0	12	6	16	0	19	11
Young female C57Bl/6	no tissue	0	6	14	6	0	9	19
