# Supplementary figures and images for: Generation and Characterization of a SARS-CoV-2-Susceptible Mouse Model Using Adeno-Associated Virus (AAV6.2FF)-Mediated Respiratory Delivery of the Human ACE2 Gene
Source: Viruses. 2022 Dec 28;15(1):85. doi: 10.3390/v15010085 (PMC9863330; doi:10.3390/v15010085)

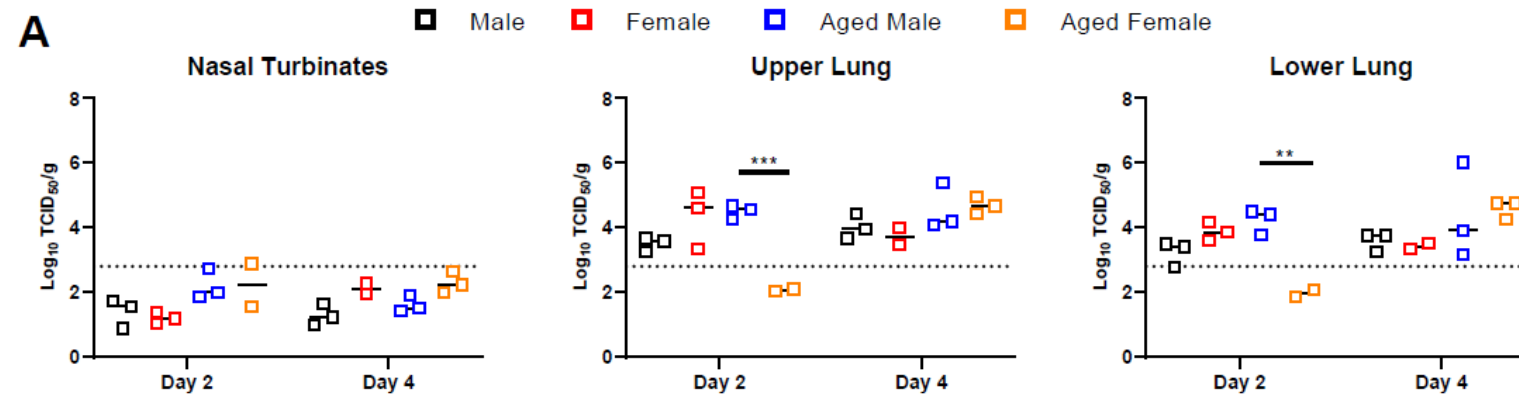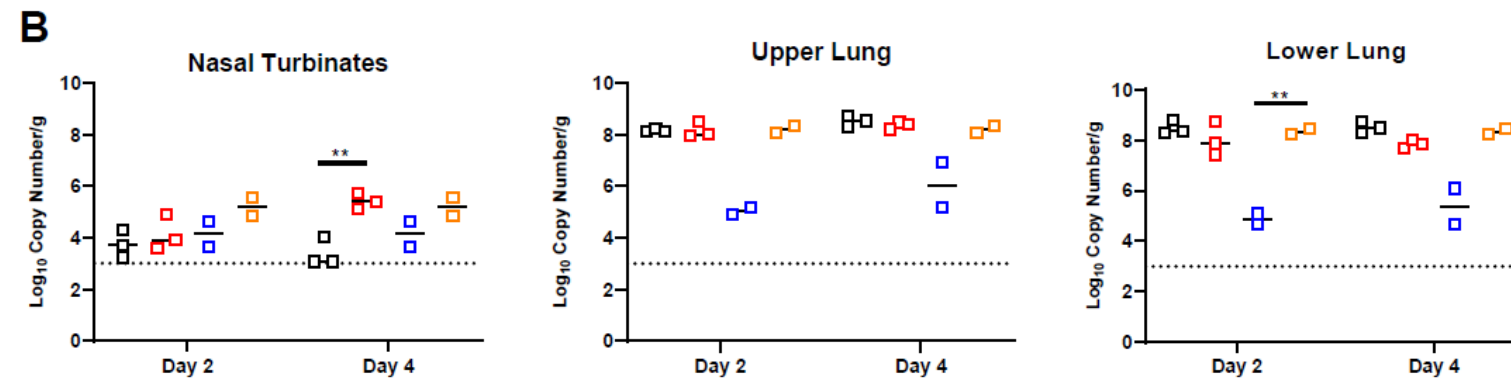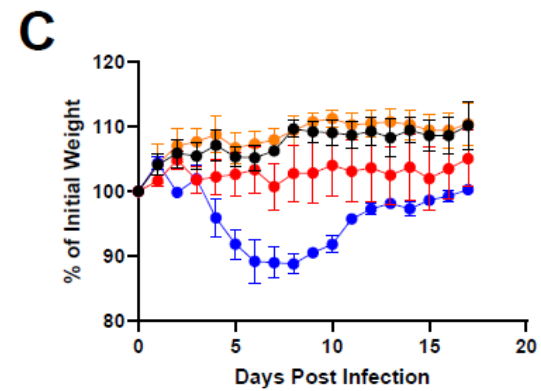

Supplement: Supplementary file 1 [file viruses-15-00085-s001.zip › Figure S1.pdf]

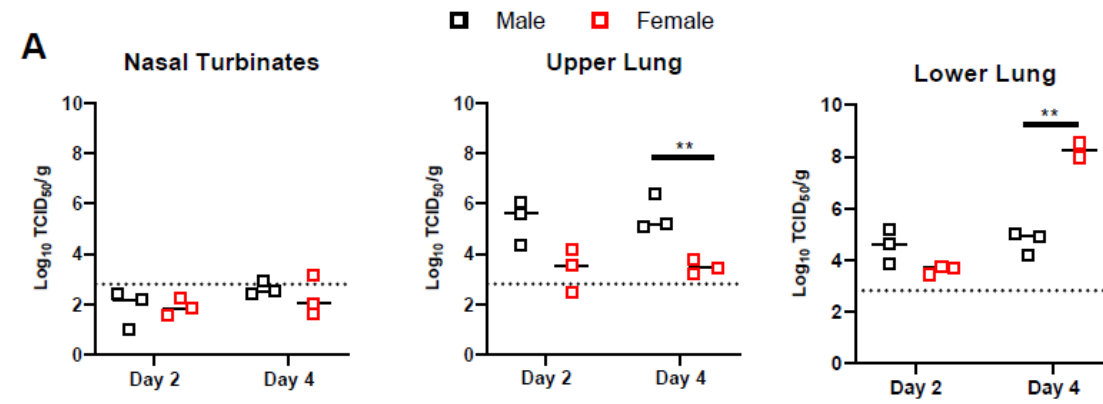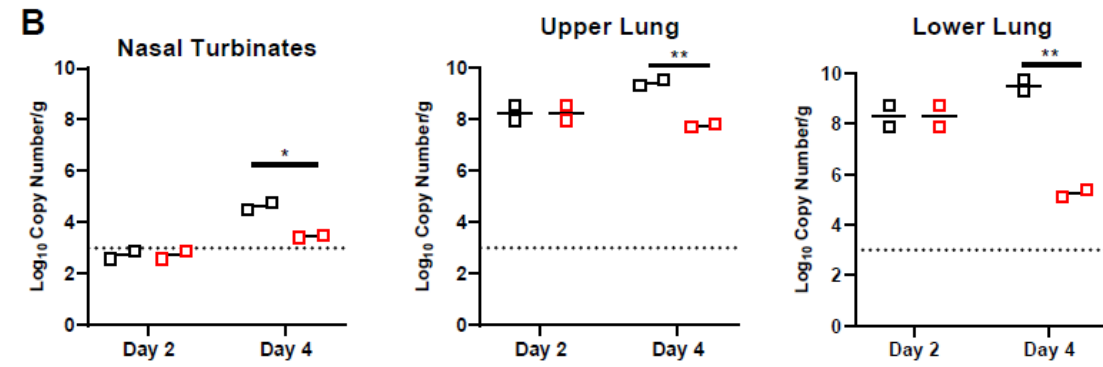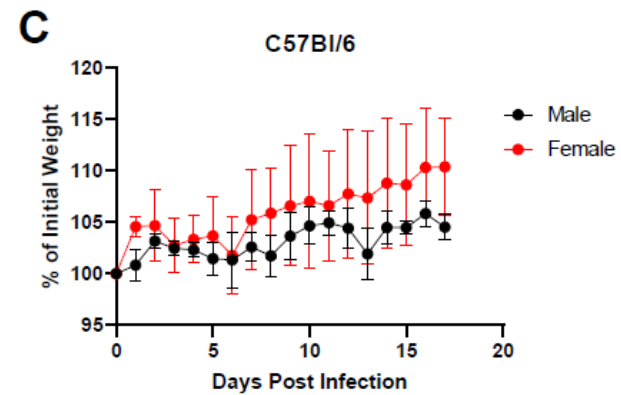

Supplement: Supplementary file 1 [file viruses-15-00085-s001.zip › Figure S2.pdf]
