# Supplementary material for: Generation and Characterization of a SARS-CoV-2-Susceptible Mouse Model Using Adeno-Associated Virus (AAV6.2FF)-Mediated Respiratory Delivery of the Human ACE2 Gene
Source: Viruses. 2022 Dec 28;15(1):85. doi: 10.3390/v15010085 (PMC9863330; doi:10.3390/v15010085)
Supplement: Supplementary file 1 [file Figure S3.pdf › Figure S3.pdf]

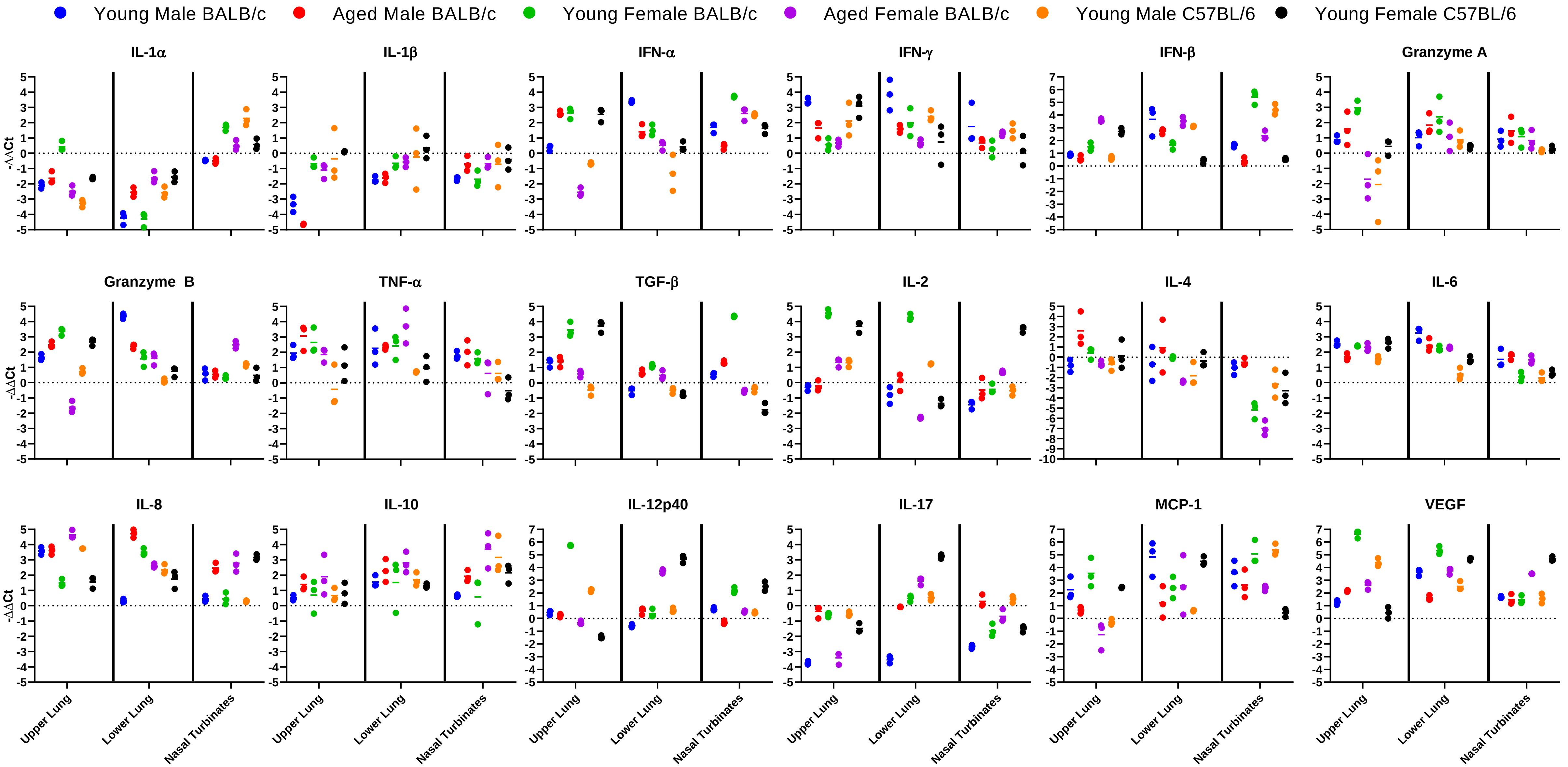

Young Male BALB/c
Aged Male BALB/c
Young Female BALB/c
Aged Female BALB/c
Young Male C57BL/6
Young Female C57BL/6
IL-1α
IL-1β
IFN-α
IFN-γ
IFN-β
Granzyme A
Granzyme B
TNF-α
TGF-β
IL-2
IL-4
IL-6
IL-8
IL-10
IL-12p40
IL-17
MCP-1
VEGF
-ΔΔCt
Upper Lung
Lower Lung
Nasal Turbinates
